# Supplementary figures and images for: Changes in calpain-2 expression during glioblastoma progression predisposes tumor cells to temozolomide resistance by minimizing DNA damage and p53-dependent apoptosis
Source: Cancer Cell Int. 2023 Mar 17;23:49. doi: 10.1186/s12935-023-02889-8 (PMC10022304; doi:10.1186/s12935-023-02889-8)

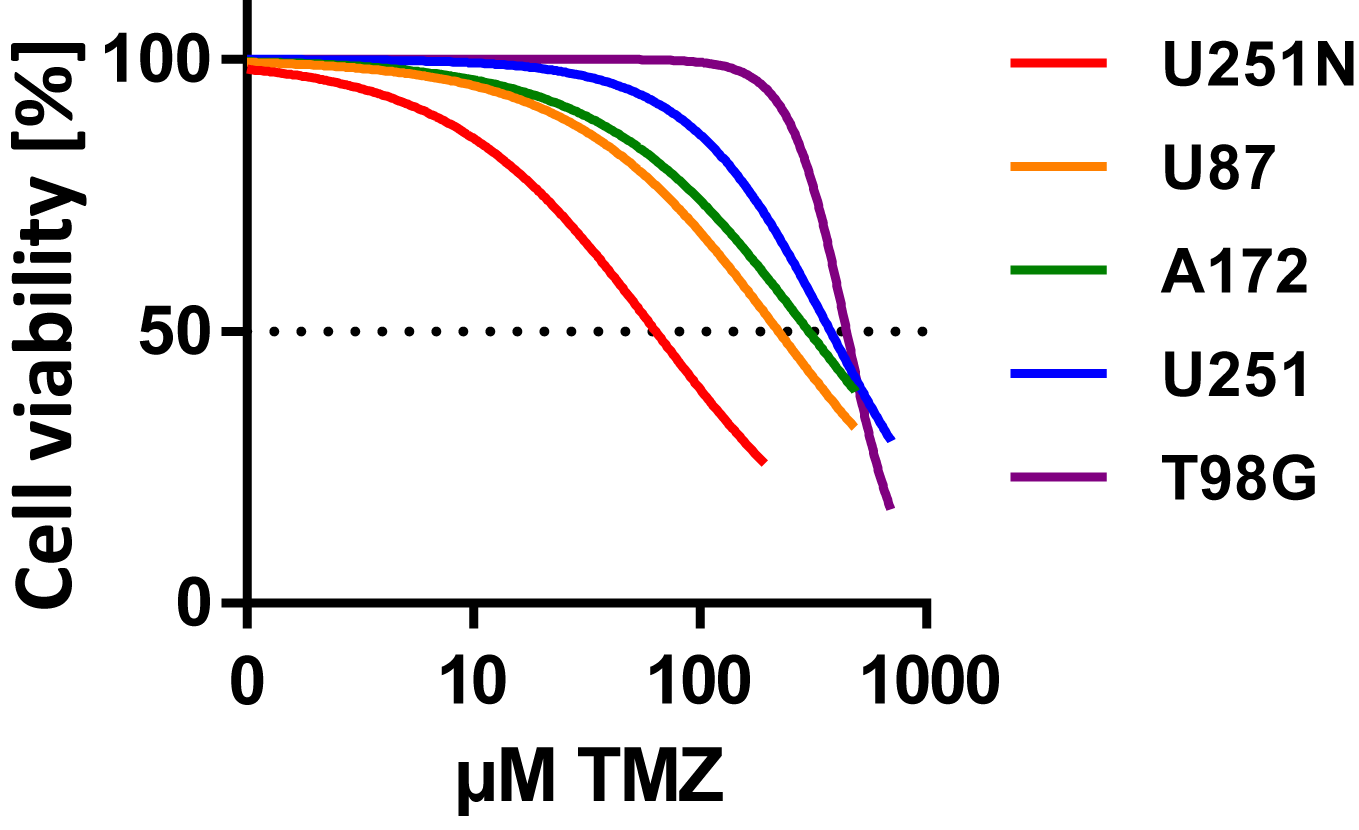

Supplement: Supplementary file 1 — Additional file 1. IC50 curves for TMZ of five established GBM cell lines. [file 12935_2023_2889_MOESM1_ESM.tif]
